# Epidemiology of emerging zoonoses in Israel.

**DOI:** 10.3201/eid0302.970221

**Published:** 1997

**Authors:** A Shimshony

**Affiliations:** Veterinary Services and Animal Health, Beit Dagan, Israel.


### Epidemiology of Emerging Zoonoses in Israel

The epizootiologic, climatic, and ecologic conditions in the Middle East, combined with socioeconomic and agricultural structures in Israel, have created a unique and hazardous veterinary public health situation in this country. In this paper, emerging zoonotic diseases are newly introduced disease agents or endemic zoonoses whose previous epidemiologic patterns have changed.

As of 1996, 15 zoonotic diseases of animals, anthrax, brucellosis, cysticercosis (bovine), erysipelas, leishmaniasis (canine), leptospirosis, listeriosis, malleus (glanders), psittacosis (chlamydiasis), Q fever, rabies, Rift Valley fever (RVF), salmonellosis, trichinosis, and tuberculosis (bovine and avian) (Table 1), have been declared notifiable in Israel. When diagnosed in animals, these diseases are officially reported to the state veterinary officers, who in turn report them to the medical officers. Of these notifiable zoonoses, RVF has never been recorded in Israel, and glanders has not been reported since 1951; ten are notifiable in humans at the national level.

This article examines five of the reportable animal diseases whose epidemiologic features have changed in Israel during the last decade: *Brucella melitensis* in cattle, *Salmonella enteritidis* in poultry, rabies, canine leishmaniasis, and trichinosis in wild boars. Two nonreportable diseases in animals—botulism in ruminants and echinococcosis—will also be discussed briefly. (Table 1). Recently, the possibility that bovine spongiform encephalopathy (BSE) may be a zoonotic disease has been discussed worldwide. Thus, we also describe steps adopted by the Veterinary Services and Animal Health (VSAH) to prevent the introduction of BSE into Israel. The data on zoonotic diseases in animals are from VSAH's annual reports to the Office International des Epizootics in Paris. If not otherwise mentioned, the data on the diseases in humans are from the weekly and monthly reports of the Ministry of Health in Jerusalem.

Table 1. Selected zoonotic diseases in Israel, 1996

| | Animal | | Human | |
|---|---|---|---|---|
| Disease | Year Notifiable | Occurrence | Year Notifiable | Occurrence |
| Anthrax | 1945 | 1993 | 1949 | 1984 |
| Brucellosis | 1952 | +++ | 1949 | + |
| Cysticercosis (bovine) | 1981 | + | NN | +... |
| Erysipelas | 1945 | (+) | NN | +... |
| Leishmaniasis (canine) | 1945 | ( ) | 1949 | 1994 |
| Leptospirosis | 1945 | + | 1949 | (+) |
| Listeriosis | 1961 | + | 1993 | +... |
| Malleus (glanders) | 1945 | 1951 | NN | never |
| Psittacosis | 1952 | + | NN | +... |
| Q fever | 1952 | + | 1949 | (+) |
| Rabies | 1955 | + | 1949 | 1960 |
| Rift Valley fever | 1978 | never | NN | never |
| Salmonellosis | 1959 | +++ | 1949 | ++ |
| Trichinosis | 1982 | ( ) | No | +... |
| Tuberculosis (bov., av.) | 1945 | 1993 | 1949 | – |
| Botulism (types C,D) | NN | + | 1993 | – |
| Echinococcosis | NN | ++ | 1980 | (+) |

NN Not notifiable
0000 Never reported
- not reported, probably no occurrence
Year Year of last occurrence
(+) exceptional occurrence
\+ low sporadic occurrence
++ enzootic/endemic
+++ High occurrence

### *Brucella melitensis* in Dairy Cattle

Bovine brucellosis is usually caused by *Brucella abortus*, less frequently by *B. melitensis*, and rarely by *B. suis*. The disease is manifested by abortion and excretion of the organisms in uterine discharges and in milk. Brucellosis is highly pathogenic for humans, *B. melitensis* is regarded as more pathogenic than *B. abortus*. *B. melitensis* is the main causative agent of caprine and ovine brucellosis; it appears to occur mainly in the Mediterranean regions, but infection occurs worldwide.

As of 1996, the dairy cattle of Israel, approximately 120,000 lactating cows and 230,000 younger animals on 1,500 farms, are free of infection with *B. abortus*. Since 1984, VSAH's ongoing, compulsory surveillance of all farms has reported no cases of *B. abortus*. The surveillance includes periodic bulk milk testing. In the event of a positive reaction, the test is repeated on bulk milk from groups of cows, and the cows of a suspected group undergo individual blood testing. Cows with positive test results are slaughtered with full state compensations, and their organs are bacteriologically examined in the State Reference Laboratory for Brucellosis in the Kimron Veterinary Institute. In spite of the absence of *B. abortus,* vaccinations of all 2- to 6-month-old dairy heifers with *B. abortus* strain 19 vaccine has not been discontinued.

The status of *B. melitensis* in small ruminants, however, is different. Brucellosis, which has been present in sheep and goat flocks (mainly in the Bedouin sector) for decades, has increased considerably since the mid-1980s A countrywide serologic survey was carried out during 1994–1995, in which 906 (10.1%) out of the 4,738 examined flocks were found infected. The total number of reacting animals was 4,785 (3.16%) out of 151,409 examined sheep and goats. Accordingly, a sharp rise in the occurrence of *B. melitensis* in humans has been recorded from one case per 100,000 in 1983 to 11 cases per 100,000 (total 498 cases) in 1988 to 11 cases per 100,000 (total 498 cases) in 1988. The most common sources of human infection are locally prepared unpasteurized goat and sheep cheeses and milk (1).

An important phenomenon regarding *B. melitensis* in Israel is its penetration into cattle herds. Until the late 1980s, these were mainly small herds of locally bred Baladi beef cattle. In sporadic exceptional cases, introduction of *B. melitensis* into dairy cattle farms was also observed. Such a case was recorded for the first time in 1977 when raw unpasteurized whey, a by-product of sheep cheese, from a central dairy plant was fed to the lactating cows in one of the largest and finest dairy herds in Israel, in the centrally situated Valley of Yezreel (2). The case came to the attention of VSAH only after three dairy workers were hospitalized with Malta fever. It took 2 years and the slaughter of 28 cows for the herd to become disease-free again. According the VSAH "test and slaughter" policy, all cattle in a farm where the seropositivity rate does not exceed 50% are subject to monthly repeated serologic examinations. Animals with positive test results are slaughtered, and compensations are paid to the owners. The farm may be declared free of brucellosis, and qua-rantine measures are discontinued after three consecutive negative serologic tests of the entire herd. If more than 50% of the cows react positively to the serologic test, the entire herd is slaughtered. Since this incident, only pasteurized whey has been permitted as animal feed; however, a second major outbreak occurred in late 1988, when cattle on a dairy farm with 765 animals in western Galilee tested positive on the periodic milk-ring test and was infected with *B. melitensis* (3). Circumstantial evidence implicated contaminated whey from sheep or goat milk as the most likely vehicle of infection. The epidemiologic investigation was inconclusive, but researchers hypothesized that the pasteurized whey could have been recontaminated when transported in tankers not properly disinfected after having transported raw goat's milk. The dairy herd was put under quarantine, and test and slaughter policies were applied; implementation of these policies led to the compulsory slaughter of 175 animals (±23% of the herd). The farm was declared free of brucellosis a year after the initial infection and two consecutive herd tests with negative results. No human cases were involved. From this outbreak, it could be concluded that vaccination of cattle with strain 19 vaccine did not confer adequate protection against infection with *B. melitensis,* although it may have prevented abortions. These observations were later supported by additional data from other outbreaks in dairy cattle.

Since 1988, eight additional outbreaks of *B. melitensis* in large dairy cattle farms throughout Israel have been recorded (Figure 1). The largest outbreak occurred in 1994 when the entire cattle population (761 cattle) of a large kibbutz dairy farm had to be slaughtered because the infection rate exceeded 60%. In this outbreak, more than 30 members of the kibbutz community, as well as the attending veterinarian, were infected, most by direct contact with the infected cows or placenta and by the consumption of unpasteurized milk. At present, an additional outbreak with human cases is being investigated.

The last eight outbreaks have been attributed to indirect contact with infected nomadic sheep and goat flocks, which apparently contaminated the pasture from which fodder was supplied to the lactating cows. Another route of infection might have been dogs, most probably infected by consuming infected placentas. Serologic examinations of dogs were carried out on two of the farms, and antibodies were found in samples of 9 of the 39 dogs tested. The infected dogs were then euthanized. It should be noted that Israel is free of *B. canis*. The introduction of *B. melitensis* into

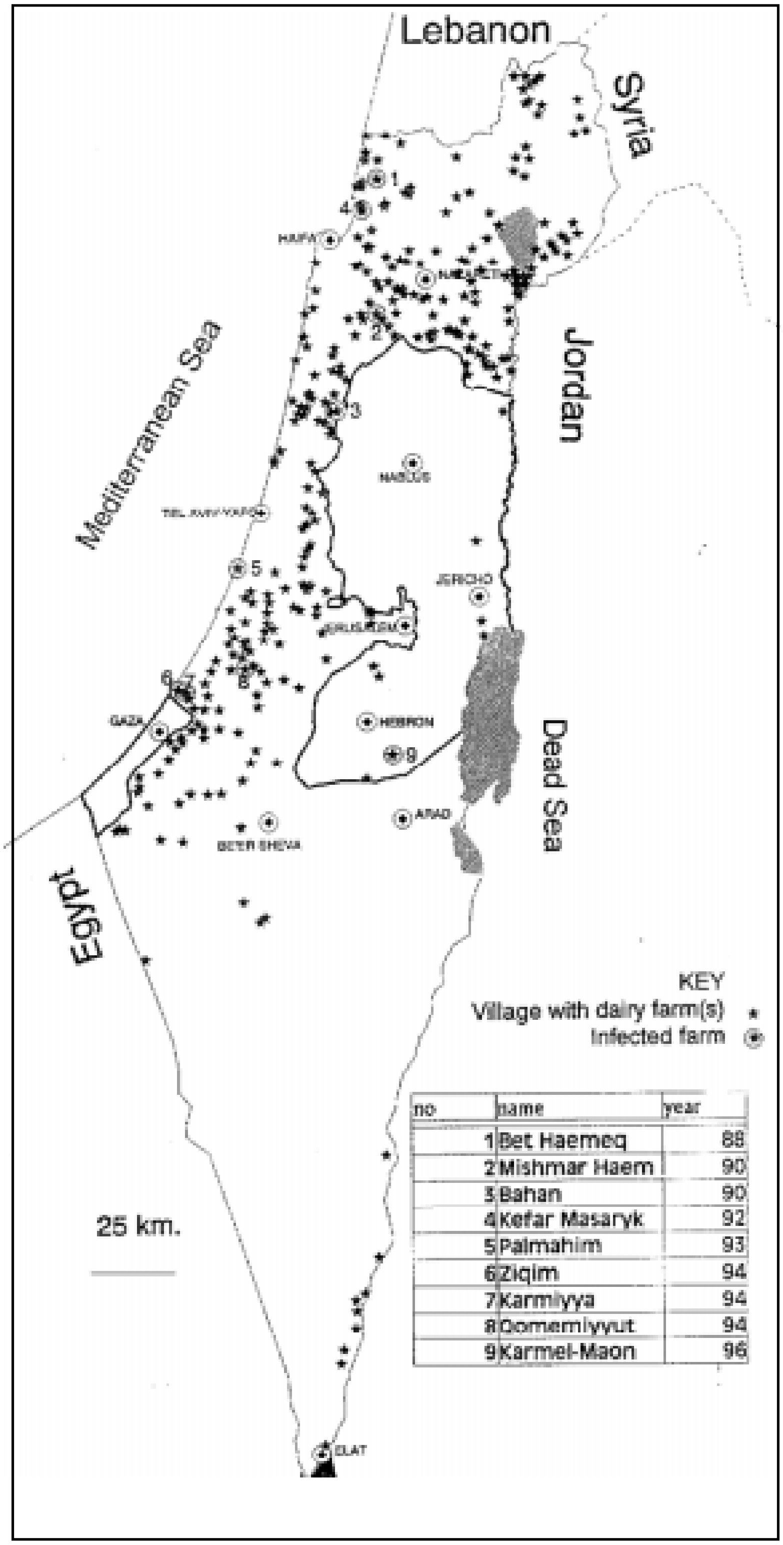

Figure 1. *Brucella mellitensis* in dairy cattle, Israel 1988–1996.

dairy cattle farms, a serious public health hazard, may be almost inevitable in a highly contaminated environment.

### *Salmonella enteritidis*

Salmonellosis is an infectious disease of humans and animals caused primarily by intestinal bacteria of which 2,375 serovars are recognized, a number constantly increasing. Salmonellosis appears to be most prevalent in areas of intensive animal husbandry, especially of poultry or pigs. *S. enteritidis*, especially of the phage-type TP4, emerged as a serious public health hazard in Europe during the late 80s, mainly as a contaminant of table eggs because of its transovarian infection in laying hens (4). National programs have been implemented in some countries to control *S. enteritidis* in poultry and protect the consumer.

Since the initial report from Europe, in 1988, of the emerging animal and public health problem caused by *S. enteritidis* PT4 in poultry, Israeli policy and activities have undergone a three-phase evolution:

1) 1988–1991: To prevent introduction of *S. enteritidis,* Israel implemented strict quarantine conditions, including testing (serologic and bacteriologic), on the import of breeding poultry, chicks, and hatching eggs. From 1988 to 1991, 11 imported breeding flocks (five broiler chickens, five laying chickens, one turkey), 103,100 birds, and 149,970 hatching (layer) eggs were found infected with *S. enteritidis* PT4 and destroyed. The policy of strict border control of imported day-old chicks and hatching eggs probably contributed to the delay in the appearance of human infection until 1992.

2) 1992–1993: For the first time local breeding flocks were found infected with *S. enteritidis* PT4 and destroyed along with their offspring laying chick flocks. A surveillance program was implemented in the 200 local breeding flocks. The program included bacteriologic examinations of dead birds forwarded to the regional poultry disease laboratory, as well as monthly visits of state poultry disease officers to each breeding farm for sanitation control that included drag swab tests, sampling of caecal contents for bacteriologic examinations, and in several flocks, rapid slight agglutinations. Hatcheries were included as well. After the surveillance, infected flocks (31 [10 broilers, 20 layer, one goose] which comprised 272,188 birds and 2.8 million hatching eggs) were destroyed. The state had to compen-sate the owners approximately 1.5 million U.S. dollars; $300,000 in 1992, $1.17 million in 1993). "Competitive exclusion," a preventive-curative technique developed in Finland and tried experimentally on six breeding flocks produced inconclusive results (5). During 1992 and 1993, a significant increase in laboratory-contracted human cases of *S. enteritidis* PT4 infection was recorded by the Ministry of Health: nine cases in 1991, 203 in 1992, and 473 in 1993.

During 1993, to evaluate the *S. enteritidis* infection rate on commercial farms, VSAH surveyed 88 broiler and layer breeding farms and found 18 (26.8%) of the 67 surveyed broiler breeder farms and 6 (28.5%) of the 21 layer breeder farms infected by direct cultures. In another investigation, 35 flocks of laying hens were examined in slaughterhouses, of which 12 (30.8%) were infected with *S. enteritidis*. Out of the said 10 isolates, five were identified as PT4. In addition, 39 hatcheries throughout the country were surveyed, of which 12 (30.8%) were infected with *S. enteritidis*. These findings, combined with the alarming increase human *S. enteritidis* infection rates, led to a reevaluation of the general policy, which examined experience in other countries and local data. One of the main decisions was to concentrate the efforts in the layer line and discontinue the stamping out policy in the broilers line. The first step was to prohibit the simultaneous incubation of layer- and broiler-hatching eggs in the same hatchery.

The updated policy to concentrate and intensify the control efforts upon the layers breeding stock while generally improving sanitary conditions in all lines and to implement the control upon handling of table-eggs seems to have contributed to the improvement of the public health situation regarding *S. enteritidis* PT4.

3) 1994–

In 1993, after the publication of state regulations forbidding the simultaneous incubation of layer and broiler hatching eggs, VSAH discontinued intensive surveillance and, eventually, stamping out of broiler breeding farms, to concentrate efforts on the layer line. Consequently, the number of stamped-out broiler poultry flocks decreased from 24 in 1993 to nine in 1994 to only one in 1995. VSAH implemented four steps: a) Strict control of the layers breeding stock and hatcheries, including bacteriologic examinations of meconium, offspring, and laying birds and monthly sampling of drag swabs from the hatcheries and the breeding farms; b) improvement of sanitary conditions in poultry farms; c) introduction of an (inactivated) *S. enteritidis* vaccine, combined with the live attenuated *Salmonella typhimurium* vaccine in parent-stock breeding flocks; and d) enforcement of new state regulations regarding the handling of table-eggs, their collection, transportation, packing, marking, chilling, and marketing.

These measures were possible because of a single, centralized veterinary system that combines modern diagnostic facilities, field activities, and legal enforcement tools. The results of the measures described are reflected in the number of reported human *S. enteritidis* isolates (Table 2).

Table 2: Reported *Salmonella enteritidis* isolates in humans

| | *S. enteritidis* | *S. enteritidis (PT4)* |
|---|---|---|
| 1989 | 357 | - |
| 1990 | 878 | - |
| 1991 | 987 | 9 |
| 1992 | 926 | 203 |
| 1993 | 1376 | 473 |
| 1994 | 1450 | 750 |
| 1995 | 997 | 557 |

Furnished by Dr. Vered Agmon, Ministry of Health, Jerusalem. Preliminary figures for 1996 indicate that decline continues.

### Rabies

Rabies, a major zoonosis caused by the neurotropic Lyssavirus, transmissible to all mammals, occurs in all parts of the world. It is especially prevalent in the temperate zones and where there is a large canine population. Generally, in countries where the virus reservoir is predominantly within the stray dogs which are the main vectors, the term "urbanic rabies" is applied. On the other hand, when the reservoir is mainly the wildlife population, the term "sylvatic rabies" is used. Urban rabies predominates in Asia, Africa, and South America.

The evolution of rabies in vector animals, since 1948 is presented in Table 3. Annual vaccination of owned dogs became compulsory in 1957; vaccination coverage is estimated at ±60%. In 1979, rabies became sylvatic (involving mainly fauna [foxes] rather than dogs) and remained so until 1990. A transitory change was observed during 1991–92 when a sharp rise in the number of rabid dogs converted the picture into "urban rabies" (dogs/fauna ratio:2/1). This change, combined with the penetration of the disease into the densely populated coastal region where rabies had been practically absent for more than 30 years, could have been an outcome of the Gulf war at the beginning of 1991 when many people (nearly 50% of the inhabitants of Tel Aviv and other coastal cities) left their homes for safer areas during the Iraqi missile attacks. Many of them abandoned their pets, establishing a large stray-dog population. Subsequent steps to eliminate stray dogs may have reversed the situation during 1992. Since 1993 (up to November 1996), the pattern of predominantly sylvatic rabies has been reestablished.

Rabies is practically endemic in most of Israel; clusters of rabies cases in foxes are observed mainly in areas around the cities of Beer-Sheva, Arad, Jerusalem, and Nazareth, but sporadic cases are scattered in other areas (Figure 2).

The wildlife potentially involved with rabies comprises mainly foxes (*Vulpes vulpes*) but in recent years, a significant increase in the number of jackals (*Canis aureus*) has been observed in the central and northern parts of the country. During 1995, 63 foxes and 14 jackals were tested at the central rabies diagnostic laboratory at the Kimron Veterinary Institute; 30 (47.6%) foxes and 2 (14.3%) jackals were infected. The figures for 1996 are similar. Other wildlife species, involved with rabies are the mongoose (*Herpestes ichneumon ichneumon*), badgers (*Meles meles canescens*), and the stone marten (*Martes foina syriaca*). During 1986 to 1996, rabies was diagnosed in 251 foxes, 31 jackals, four mongooses, four badgers, and three martens, which were sent to the Kimron Veterinary Institute. The actual incidence of the disease was much higher. In view of the growth of the jackal population, rabies—if spread within that species—may increase considerably the already serious threat to humans and farm animals. Recently, seven cattle herds in northern Israel were found infected, more than twice the mean annual number for decades. This might reflect the increased rabies incidence among the extremely dense populations of foxes and jackals in that region.

Oral vaccination of wildlife in Israel has been considered, but vaccine and baits evaluation is still in its experimental stages. At any rate, vaccination will only be practical if it is effective for both foxes and jackals and involves entire ecologically related regions (the West Bank, the Jordan Valley, and probably the western parts of Jordan and southern Lebanon).

### Canine Leishmaniasis

Canine leishmaniasis is a chronic viscera-cutaneous disease caused by *Leishmania infantum* (in the Old World), of which the dog acts as the source reservoir. The vectors of leishmaniasis are phlebotomine sandflies belonging to the genera Phlebotomus (Old World) and Lutzomyia (New World).

Canine leishmaniasis was common before 1948 but seemed to disappear after that. Since 1955, no cases have been reported in dogs. However, four human cases of visceral leishmaniasis (Kala-azar) (two in 1980 and two in 1982) were reported by the Ministry of Health in two restricted areas in eastern and western Galilee. The disease became regarded as endemic in these areas, although no canine cases were detected there.

Table 3. Mean annual number of rabies cases in animals and humans, 1948–1995

| Years | No. of years | Domestic animals Dogs | Cats | Wildlife Foxes | Jackals | Other fauna | Fauna (%) | Human cases |
|---|---|---|---|---|---|---|---|---|
| 1948-1957 | 10 | 72.0 | 3.9 | 0.1 | 9.9 | 0.4 | 12.0 | 23 |
| 1958-1966 | 9 | 19.4 | 0.9 | 0.3 | 0.8 | 0.1 | 5.6 | 2 |
| 1967-1978 | 12 | 8.6 | 0.3 | 1.3 | 0.3 | 0.16 | 16.5 | 2 |
| 1979-1990 | 12 | 6.2 | 0.25 | 12.6 | 1.25 | 1.3 | 70.1 | 0 |
| 1991-1992 | 2 | 27.0 | 0 | 11.0 | 2 | 0 | 32.5 | 0 |
| 1993-1995 | 3 | 14.25 | 1.25 | 35.25 | 4 | 4 | 72.2 | 0 |

From January 1995 through November 1996, 30 clinical cases of canine leishmaniasis were reported from five communities in the Judean hills northwest of Jerusalem and from one location in western Galilee (Figure 3). Diagnosis was carried out by the Koret Veterinary School and the Kuvin Center of the Hebrew University, which reported that the cases were caused by *L. infantum* (G. Benet, pers. comm.).

### Trichinosis

Trichinosis is a parasitic disease. Human infections are established through the consumption of insufficiently cooked pork infected with the causative nematode *Trichinella spiralis*.

No cases of trichinellosis (trichinosis) have been recorded in domestic pigs in Israel since 1948, though a substantial number of pigs are slaughtered (more than 100,000 in 1995) and each batch is sampled for representative microscopy examinations. In light of the accumulated data about the absence of infection in the housed pigs, the sample rate is limited. Trichinosis in animals was declared notifiable in 1982 when its potential hazard was reflected by the admission to Israeli hospitals of 20 clinically infected persons from southern Lebanon, close to the northern Israeli border.

According to a state regulation published in 1977, the entire carcass of hunted wild boars should be presented to accredited veterinary inspectors at specified inspection stations. Until 1991, 100–200 such carcasses, approximately 10% of the actual number of killed boars, were annually presented for microscopy examinations without detecting any infections. However, in 1992, hunters from northern Galilee were hos-pitalized with symptoms of acute trichinosis after eating pork derived from game. Consequently,

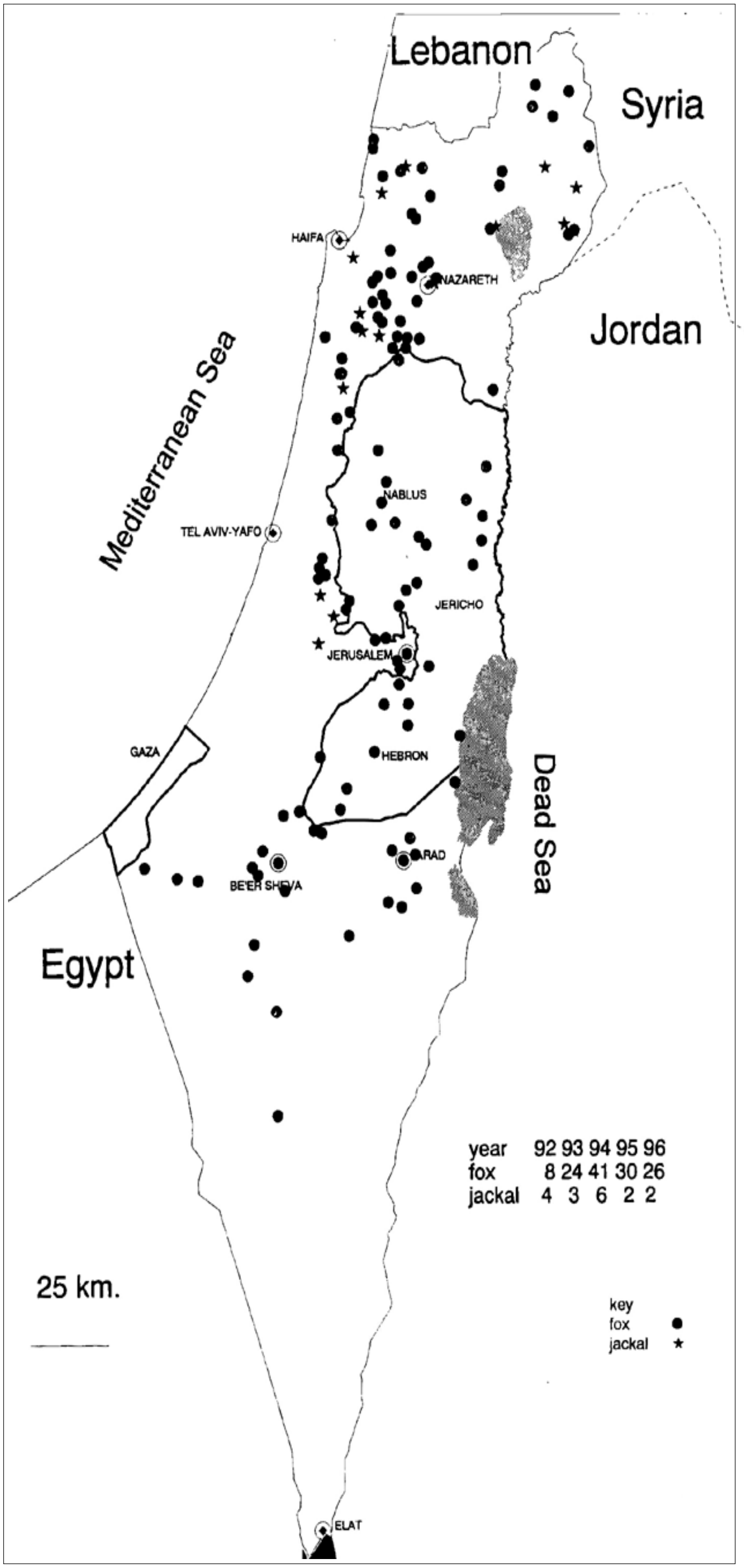

Figure 2. Rabies in fauna in Israel, 1992–1996.

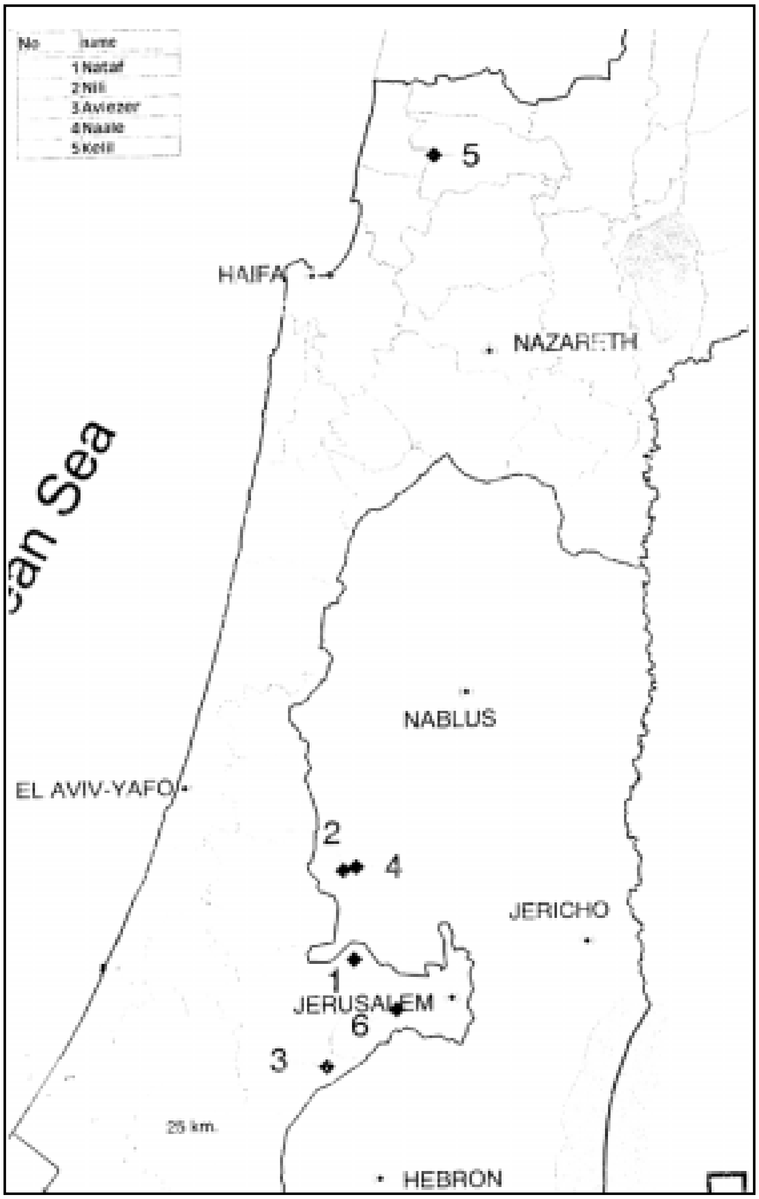

Figure 3. Canine Leishmaniasis in Israel, 1995–1996.

public awareness of the disease grew, and the number of samples from wild boars submitted for laboratory examination increased two- to three-fold from previous years. In May 1992, the first case was found in a wild boar from the Lebanese frontier, followed by 19 additional cases be-tween 1993 and 1996, all in wild boars shot in three areas: (upper Galilee; central and western Galilee, and the Carmel Hills). By October 1996 alone, no fewer than nine cases of trichinosis were diagnosed.[1] Accor-ding to the Animal Diseases Regu-lations, carcasses of animals found infected are condemned; no alter-native measures, such as cooking or freezing, are officially permitted.

### Botulism

Botulism is a highly fatal toxemia caused by ingestion of the toxin of *Clostridium botulinum*. There are eight types and subtypes of *Cl. botulinum*, of which types A, B, and E are of most importance in human botulism; types C and D affect farm animals, mainly cattle, horses, chickens, and sheep.

In 1978, a major outbreak of botu-lism (type D) caused the deaths of many dairy cattle and small rumi-nants from farms in central and nor-thern Israel (7). Spores of *Cl. botu-linum* type D, as well as the toxin itself, spread to many farms from two central animal feed plants by recycled poultry manure that included remains of avian cadavers

[1]Dr. John Wortabet (1827–1908), an Armenian-Lebanese physician who worked in the St. John Hospital in Beirut and lectured in the medical college there during the second half of the 19th century diagnosed trichinosis in humans in south Lebanon during a massive outbreak in the village of El-Chiam in November 1880—more than 115 years ago. His exemplary observations about the outbreak, which involved 262 cases, including six deaths, were published in Lancet (6). A second outbreak, in the Northern Golan, was reported by Dr. Wortabet in Lancet (4 August 1883). In this outbreak, 40 inhabitants of the village Ein-Kinya, were infected after consuming raw pork from a wild boar. However, in this outbreak, no deaths were recorded. His footnote, at the end of the report, is still timely: "From personal observation and experience I have found the use of pork in Syria decidedly unhealthy. The wild boar in winter is a delicacy, but unless previously examined with the microscope, according to German law, or cooked more thoroughly than is usually done, its use cannot be free from the danger of communicating trichinae to man." Data about Dr. Wortabet courtesy of Drs. Mertyn Malkinson and Arieh Sheskin.

and was not properly sterilized. Many farms are suspected to have been contaminated by the spores through the centrally produced and countrywide distributed infected feed. Consequently, annual botulism vaccinations of cattle and of other vulnerable animals with a vaccine, including toxoids of *Cl. botulinum* types C and D adsorbed onto aluminum hydroxide gel, are conducted in most parts of Israel. Sporadic cases involving unvaccinated animals (mainly young cows) occur every year, along with an exceptionally large number of outbreaks, (23) recorded in 1995, found by the Kimron Veterinary Institute to be caused by botulism type D. In all these cases, poultry manure was the suspected source of intoxication.

Although botulism types C and D are not generally regarded pathogenic to humans, it has recently been decided to prohibit the release of meat from ruminants with suspected cases for human consumption.

### Echinococcosis

Echinococcosis, also called hydatid disease, is caused in humans by ingestion of eggs of the cestode Echinococcus, of which the most predominant and epidemiologically significant is *Echinococcus granulosus*. The adult tapeworm is carried by dogs and other carnivores. The intermediate hosts in which the cystic forming larval stage develops include various food animals and humans. The infection cycle is maintained by the availability of infected organs to carnivore hosts. Data from slaughterhouses show that the countrywide infection rate per 1,000 animals in 1995 was 0.9 for cattle and 39.9 for sheep (8).

In 1981, human hydatid disease was declared notifiable by the Ministry of Health. During 1991 to 1995, 38 human cases were officially recorded in the country, but the actual number is much higher. For example, El-On et al. (9) conducted epidemiologic studies in 1988 in the town of Yirka, a semirural Druze community of 8,200 persons in northern Israel, and found a cumulative percentage of confirmed present or recent past hydatid infections of 12 (1.6%) of 758, leading to an extrapolated rate of 1,583 per 100,000 persons, which is comparable with that in hydatidosis-endemic areas worldwide. This remarkable situation could be explained by four combined factors: 1) High infection rates in sheep and goats (±10%); 2) high infection rates in owned dogs (14.2%); 3) widespread practice of illegal slaughter of sheep and goats, performed without veterinary inspection and followed by uncontrolled disposal of the infected slaughter offal; and 4) a large number of hunting dogs kept at the homes of many of the Druze people, who traditionally hunt.

The prevalence of hydatid disease in neighboring Moslem villages was lower, mainly because of the smaller number of dogs kept at homes and the general aversion in the Moslem community to any contact with dogs.

### Israel's Measures to Prevent the Introduction of BSE

BSE, a fatal neurologic disease of adult cattle first discovered in the United Kingdom in 1986, is one of the transmissible spongiform encephalopathies caused by unconventional agents extremely resistant to heat and chemical treatments.

Israel has adopted the following rigorous steps (taken since the very first announcement of the new disease in the United Kingdom) to prevent the introduction of BSE and to safeguard early detection of eventual occurrence of the disease in the local livestock:

1. State veterinarians, farm animal practitioners, slaughterhouse veterinarians, and cattle breeders associations were notified and updated by a) articles and updates in the monthly Veterinary Bulletins (more than 350 items since December, 1987; b) workshops and lectures, central and regional, some of them with participation of British experts and demonstration of British videotaped clinical BSE cases; circulars, including detailed descriptions of clinical BSE epidemiology and symptoms.

2. The import of meat and bone meals, derived from ruminants from the United Kingdom was banned since December 1988, and from all other countries since July 1990.

3. BSE was declared an officially notifiable disease in 1992.

4. A pathologist from the Kimron Veterinary Institute was trained in contemporary diagnostic techniques at the Central Veterinary Laboratory, Weybridge, United Kingdom (October 1992).

5. Since 1993, bovine brains presented to the Kimron Veterinary Institute after any CNS-related symptoms, are histopathologically examined for BSE changes. During 1993–1996, 583 brains (1993, 72; 1994, 134; 1995, 167; 1996, 210) were examined, all found negative. Most of the brains were of bovines older than 2 years. This sample size exceeds the number of brains to be examined annually to detect BSE on a country level, as recently recommended by the Office International des Epizootics. The recommendation was to examine a minimum of 50 brains annually if the national cattle population, 209 months of age or older, is up to 500,000 and 91 brains if the cattle population is 1,000,000. The Israeli cattle population of said age group is less than 250,000.

6. In April 1996, an interministerial BSE expert advisory panel was established to recommend new/updated steps for BSE prevention/ monitoring.Detailed recommendations were presented in July 1996, most of which are already adopted.

7. The panel paid special attention to the import conditions for meat and offals. This issue is related to the documented presence in Israel, of a considerable population-cluster that may be exceptionally vulnerable to transmissible spongiform encephalopathies. Accumulated scientific evidence indicates that this population, composed mainly of Jewish persons of Libyan and Tunisian origin, demonstrates a genetically linked susceptibility to CJD, probably related to a codon 200 mutation (10). This is expressed by an annual CJD incidence of nearly 100 per million, compared to less than 1 per million in the general population (E. Kahana, pers. comm.). Probable enhanced vulnerability of these persons, if exposed to the BSE agent, cannot be ruled out.

8. Another recommendation referred to adding immunohistochemistry and immunoblotting techniques to the routinely used conservative histopathologic examination of BSE and further increasing the number of examined bovines.

9. CJD was declared a notifiable disease in humans by the Ministry of Health in June 1996.

10. Since August 1996, the feeding of farm animals, including poultry and fish, with meat and bone meals derived from mammals has been officially banned.

11. Imported meals from poultry or fish origin are systematically examined at the port of entry to confirm the obtained written certification regarding the absence of mammalian ingredients.

The emergence of zoonotic diseases in Israel may be due to unique epizootiologic, climatic, and ecologic conditions in the regions as well as specific conditions within the country.

Nomadism, prevalence of disease carriers and their vectors, close proximity of ultramodern farms housing susceptible livestock of exotic breeds or their crosses to traditionally maintained flocks of disease-endemic breeds, and dense overpopulated protected wildlife are the major contributing factors to emerging zoonoses. But the cardinal factors predominant in the Middle East are the need for improved veterinary infrastructure, direct information exchange, and cooperation between the countries of the region.

Out of the seven diseases we reviewed, at least four, brucellosis, rabies, leishmaniasis, and echinococcosis, can be effectively controlled only if cooperation and animal health information systems are established on a regional level.

The assistance of the international community to the establishment of regional veterinary cooperation in the Middle East is of great importance.

**Acknowledgements**

Thanks to Dr. Van Ham of the Department of Epidemiology, Veterinary Services and Animal Health, Beit Dagan, Israel, for the maps used in this article.

**Arnon Shimshony**
Veterinary Services and Animal Health,
Beit Dagan, Israel

### References

1. Slater PE, Costin C, Seidenbaum M, Ever-Hadany S. Epidemiology of human brucellosis in Israel. Public Health Reviews, 1992;18:159-60.
2. Shimshony A. Activities of the Israeli Veterinary Services, 1973-1982. Refuah Vet 1983;40:143-203.
3. Davidson M, Shimshony A, Adler H, Banai M, Cohen A. Protection of brucellosis-free areas from reinfection. In: Adams LG, editor. Advances in brucellosis research. College Station (TX): Texas A&M University Press, 1990;400-22.
4. Hopper SA, Mawer S. Salmonella enteritidis in a commercial layer flock. Vet Rec 1988;123:351.
5. Nurmi E, Nutto L, Shnetz C. The competitive exclusion concept: development and future. Int J Food Microbiol 1992;15:237-240.
6. Wortabet J. An outbreak of trichinosis (?) from eating the flesh of a wild boar. Lancet 1881;454-5.
7. Egyed MD, Shlosberg A, Klopfer U, Nobel TA, Mayer E. Mass outbreaks of botulism in ruminants associated with ingestion of feed containing poultry waste: I. Clinical and laboratory investigation. Refuah Vet

1978;35:93-9.
8. Davidson M, editor. Annual report of VSAH for 1995 (in Hebrew); Ministry of Agriculture and Rural Development, Beit-Dagan 50250, 1996;114-124.
9. Nahmias J, Goldsmith R, Schantz P, Siman M, El-On J. High prevalence of human hydatid disease (echinococcosis) in communities in northern Israel: epidemiologic studies in the town of yirka. Acta Tropica 1991;50:1-10.
10. Gabizon R, Kahana E, Hsiao K, Prusiner SW, Meiner Z. Inherited prion disease in Lybian Jews. In: Prusiner SW, et al., editors. Prion diseases of humans and animals. London, England: Ellis Horwood Ltd, 1992:168-179.
